# Decrypting the environmental sources of *Mycobacterium canettii* by high-throughput biochemical profiling

**DOI:** 10.1371/journal.pone.0222078

**Published:** 2019-09-03

**Authors:** Ahmed Loukil, Fériel Bouzid, Djaltou Aboubaker Osman, Michel Drancourt

**Affiliations:** 1 Aix-Marseille Univ., IRD, MEPHI, IHU Méditerranée-Infection, Marseille, France; 2 Université de Gafsa, Faculté des Sciences de Gafsa, Gafsa, Tunisia; 3 Institut de Recherche Médicinale, Centre d’Etudes et de Recherche de Djibouti (CERD), Djibouti, République de Djibouti; 4 IHU Méditerranée Infection, Marseille, France; New Jersey Medical School, Rutgers University, UNITED STATES

## Abstract

*Mycobacterium canettii* is a smooth bacillus related to the *Mycobacterium tuberculosis* complex. It causes lymph nodes and pulmonary tuberculosis in patients living in countries of the Horn of Africa, including Djibouti. The environmental reservoirs of *M*. *canettii* are still unknown. We aimed to further decrypt these potential reservoirs by using an original approach of High-Throughput Carbon and Azote Substrate Profiling. The Biolog Phenotype profiling was performed on six clinical strains of *M*. *canettii* and one *M*. *tuberculosis* strain was used as a positive control. The experiments were duplicated and authenticated by negative controls. While *M*. *tuberculosis* metabolized 22/190 (11%) carbon substrates and 3/95 (3%) nitrogen substrates, 17/190 (8.9%) carbon substrates and three nitrogen substrates were metabolized by the six *M*. *canettii* strains forming the so-called corebiologome. A total at 16 carbon substrates and three nitrogen substrates were metabolized in common by *M*. *tuberculosis* and the six *M*. *canettii strains*. Moreover, at least one *M*. *canettii* strain metabolized 36/190 (19%) carbon substrates and 3/95 (3%) nitrogen substrates for a total of 39/285 (13%) substrates. Classifying these carbon and nitrogen substrates into ten potential environmental sources (plants, fruits and vegetables, bacteria, algae, fungi, nematodes, mollusks, mammals, insects and inanimate environment) significantly associated carbon and nitrogen substrates metabolized by at least one *M*. *canettii* strain with plants (*p* = 0.006). These results suggest that some plants endemic in the Horn of Africa may serve as ecological niches for *M*. *canettii*. Further ethnobotanical studies will indicate plant usages by local populations, then guiding field microbiological investigations in order to prove the definite environmental reservoirs of this opportunistic tuberculous pathogen.

## Introduction

*Mycobacterium canettii* is a smooth tubercle bacillus isolated by G. Canetti in 1969 and belonging to the *Mycobacterium tuberculosis* complex [1]. About one hundred cases only have been reported in the literature, and most of these patients have been infected in the Horn of Africa and particularly in Djibouti [2, 3]. Recently, we confirmed that *M*. *canettii* was still circulating in the Horn of Africa by isolating *M*. *canettii* strains from patients presenting with pulmonary tuberculosis in Djibouti [4]. Initial molecular analysis of a large collection of *M*. *canettii* isolates revealed a high genetic diversity with traces of intraspecies horizontal gene transfer [5]. Moreover, whole genome sequencing analysis of five representative isolates showed that *M*. *canettii* was the *M*. *tuberculosis* complex species most closely related to the last common progenitor of this complex [6].

*M*. *canettii* is acknowledged as a *M*. *tuberculosis* complex species with an environmental reservoir, and this hypothesis is mainly based on the observation of the absence of human-to-human transmission [7]. However, the putative environmental sources of infection and reservoirs remain completely elusive [3, 7]. A mouse model using an oral route for *M*. *canettii* inoculation has demonstrated the potential of ingested *M*. *canettii* bacilli to relocate to lungs and other organs [8]. This experimental data supported that people could get infected with environmental *M*. *canettii* by ingesting *M*. *canettii*-contaminated drinks or foodstuffs.

In a previous study, we used the Biolog Phenotype profiling (Biolog Inc., Hayward, CA) to characterize the metabolic profile of another environmental, non-tuberculous mycobacterium, *Mycobacterium ulcerans* [9]. In that study, we defined *M*. *ulcerans* sole-carbon-source utilization profile and we found this approach particularly conclusive for the quest of environmental sources of *M*. *ulcerans*. Therefore, we embarked in using high-throughput carbon and nitrogen substrate profiling of *M*. *canettii* to help in the quest of its potential sources and reservoirs.

## Materials and methods

### Bacterial strains

*M*. *canettii* CIP 140010059^T^, *M*. *canettii* DJ480, *M*. *canettii* DJ734, *M*. *canettii* DJ514, *M*. *canettii* DJ517 and *M*. *canettii* DJ613 were isolated from clinical sources in Djibouti in 2016 [4] and *M*. *tuberculosis* Beijing family was used as a positive control [10]. They were grown at 37°C in Middlebrook 7H10 agar medium (Becton Dickinson, Le Pont de Claix, France) supplemented with 10% oleic acid-albumin-dextrose-catalase (OADC) (Becton Dickinson) and 0.5% glycerol in a biosafety level 3 laboratory.

### Biolog phenotype profiling

The Biolog Phenotype profiling (Biolog Inc.) was performed using PM1, PM2A and PM3B biolog 96-well microplates giving 190 carbon sources and 95 nitrogen sources, as previously described [9–11]. All experiments were conducted in duplicate using two independent plates during the same day for each strain. For each mycobacterial strain, colonies were removed from the Middlebrook 7H10 agar plate using a sterile swab already dipped in 0.1% Tween 80 solution, then rubbed and grinded against the wall of a dry glass tube. Colonies were starved in phosphate buffered saline (PBS) for 24 hours at 25°C in order to minimize the background in the control well without substrate sources and to prevent color development in wells with substrate sources. A suspension made in the inoculating fluid IF-0a GN/GP (Biolog Inc.) was vigorously vortexed in the presence of glass beads to declump mycobacterial cells to obtain a uniform suspension. The turbidity of the suspension was adjusted to 81% transmittance. The PM-additive solutions for each plate were made according to the manufacturer's instructions (Table 1). A mixture solution specific for each plate was prepared by mixing 1.76 mL of the mycobacterial suspension with 22.24 mL of specific inoculating fluid (Table 2) for PM1 and PM2A plates. Then, 3.52 mL of cell suspension was mixed with 44.48 mL of inoculating fluid for the PM3B plate (Table 2). Finally, the PM plates were inoculated with 100 μL of the specific mixture solution and incubated in the OmniLog (Biolog, Inc.) system at 37°C for seven days [10]. The principle of this system is based on a redox reaction from which mycobacteria reduce a tetrazolium dye to a purple color substrate. The variable level of purple color depends upon the conditions in each PM well and indicates the metabolically active state in some wells but not others. Using the OmniLog PM Software, the color change was read and recorded every 15 minutes giving a dye reduction value. The set of values obtained of each well provides kinetic information as Area Under the Curve (AUC). For each PM plate, non-inoculated wells containing only additive solutions were used as negative controls. Then, the reproducibility of the results was assessed using the OmniLog PM Software by superposing the AUCs obtained for the homologous wells of the duplicate. Then, the “mode average” calculated average AUC for the two homologous wells. The AUC of the negative controls were compared to values of positive and negative wells to set up a threshold. Wells were considered as having a moderately positive growth when the AUC of the well was equal to or lower than 1.25 times the AUC of the negative control and a highly positive growth when the AUC of the well was equal to or higher than 1.50 times the AUC value of the negative control [9].

**Table 1:** Composition and preparation of 12x PM additive solutions.

Ingredient	1x conc.	120x conc.	Grams /100 mL	PM 1 & 2 (mL)	PM 3 (mL)
MgCl_2_, 6H_2_O	2 mM	240 mM	4.88	10	10
CaCl_2_, 2H_2_O	1 mM	120 mM	1.76		
Tween 80	0.01%	1.2%	1.2	10	10
D-glucose	5 mM	600 mM	10.8	-	10
Sterile water				80	70
Total				100	100

**Table 2:** Recipe for 1x PM inoculating fluids from stock solutions for two plates of PM1, PM2 or PM3 (100 μl / well).

PM stock solution	PM (mL)
IF-0a GN/GP (1.2x)	20
PM additive (12x)	2
Dye mix G (100x)	0.24
Cell suspension (13.64x)	1.76
Total	24

### Environmental sources of metabolized substrates

Based on our previously reported study [9], we sorted all the substrates present in the PM1, PM2 and PM3B plates into 10 categories of potential environmental sources including plants, fruits and vegetables, bacteria, algae, fungi, nematodes, mollusks, mammals, insects and inanimate environment. We compared the proportion of each category to substrates metabolized by at least one of the six *M*. *canettii* strains versus substrates not metabolized by any of the six *M*. *canettii* strains using Chi-square test (R-studio) (https://www.rstudio.com).

## Results

### Substrates metabolized by *M*. *canettii* strains

All the experiments for the six *M*. *canettii* strains and *M*. *tuberculosis* were duplicated. In addition, negative wells (containing cell-free reagents) presented very low background and flat lines in all the PMs plates consistent with the absence of metabolic activity. For the positive control *M*. *tuberculosis*, 22/190 (11%) carbon substrates and 3/95 (3%) nitrogen substrates were metabolized (Table 3). For *M*. *canettii*, 17 carbon substrates and three nitrogen substrates were metabolized by the six *M*. *canettii* strains under investigation, forming the so-called corebiologome (Table 3). In addition, 36/190 (19%) carbon substrates and 3/95 (3%) nitrogen substrates for a total of 39/285 (13%) substrates here investigated were metabolized by at least one of the six *M*. *canettii* strains.

**Table 3:** Carbone and nitrogen substrates metabolized by six *M*. *canettii* strains compared with one *M*. *tuberculosis* strain Beijing on Biolog PM1, PM2 and PM3B plates.

Substrate	*M*. *canettii* CIPT59	*M*. *canettii* DJ480	*M*. *canettii* DJ734	*M*. *canettii* DJ514	*M*. *canettii* DJ517	*M*. *canettii* DJ613	*M*. *tuberculosis* Beijing
**PM1 MicroPlate™ CarbonSources**							
L-Arabinose							
D-Xylose							
D-Ribose							
L-Lyxose							
Tween 80							
L-Rhamnose							X
Acetic Acid							
L-Asparagine							
D-Trehalose							
D-Mannose							
D-Fructose-6- Phosphate							
D-Glucose-6- Phosphate							
D-Serine							
Glycyl-L-Aspartic Acid							
D-Threonine							
Glycyl-LGlutamic Acid							
Pyruvic Acid							
Acetoacetic Acid							
Mono Methyl Succinate							
Tween 20							
Tween 40							
**PM2A MicroPlate™ CarbonSources**							
D-Arabinose							
2-Deoxy-DRibose							
Palatinose							
D-Glucosamine							
5-Keto-DGluconic Acid							
Oxalomalic Acid							
Sorbic Acid							
Dihydroxy Acetone							
D-Tagatose							
3-0-β-D-Galactopyranosyl-DArabinose							
2,3-Butanediol							X
D-Raffinose							
Salicin							
Gentiobiose							
D-Fucose							
L-Pyroglutamic Acid							
Sec-Butylamine							
**PM3B MicroPlate™ NitrogenSources**							
L-Tyrosine							
D-Mannosamine							
Alloxan							

Substrates metabolized by all tested *M*. *canettii* strains forming corbiologome are shaded red. Substrates metabolized by at least one *M*. *canettii* strains are shaded yellow. Highly positive wells are shaded dark green. Moderately positive wells are shaded light green. X, substrates which are not metabolized by *M*. *tuberculosis* and metabolized by all tested *M*. *canettii*.

Comparing *M*. *canettii* with *M*. *tuberculosis*, 16 carbon substrates and three nitrogen substrates were metabolized in common by *M*. *tuberculosis* and the six *M*.*canettii* strains (Table 3). Further, the sixteen carbon substrates metabolized by at least 1/6 *M*. *canettii* strains and not metabolized by *M*. *tuberculosis* included L-rhamnose, D-trehalose, D-mannose, D-fructose-6-phosphate, 2,3-butanediol, D-fucose, D-glucose-6-phosphate, D-serine, glycyl-L-aspartic acid, D-threonine, glycyl-l-glutamic acid, pyruvic acid, acetoacetic acid, mono methylsuccinate, L-pyroglutamic acid and sec-butylamine. No difference was observed for nitrogen substrates. Among these 16 substrates, 2,3-butanediol and L-rhamnose were metabolized by the six *M*. *canettii* strains and not by *M*. *tuberculosis*.

### Environmental sources of metabolized substrates

All the 190 carbon substrates and 95 nitrogen substrates included in the PM1, PM2 and PM3B plates were classified into ten potential environmental sources. In a first step, comparison of these ten categories to carbon substrates metabolized by at least one of the six tested *M*. *canettii* strains versus non-metabolized carbon substrates yielded a significant association between *M*. *canettii* metabolized carbon sources and plants (p-value = 0.015). Further associations did not reach the 0.05 level of significance, i.e. algae (p = 0.062), mollusks (p = 0.072), insects (p = 0.096), inanimate environment (p = 0.328), fruits and vegetables (p = 0.399), bacteria (p = 0.577), fungi (p = 0.651), mammals (p = 0.7) and nematodes (p = 0.754). In a second step, comparing categories of potential sources to the carbon and nitrogen substrates metabolized or not by at least one *M*. *canettii* strain confirmed a significant association with plants (p = 0.006). While the associations with insects (p = 0.051) were marginally significant, the associations with algae (p = 0.072), fungi (p = 0.1525), mollusks (p = 0.355), inanimate environment (p = 0.4165), nematodes (p = 0.5072), fruits and vegetables (p = 0.5176), bacteria (p = 0.783) and mammals (p = 0.8668) were not statistically significant.

## Discussion

We are reporting on the high throughput biochemical profiling of *M*. *canettii* using the OmniLog® system (Biolog Inc.). This approach has already been reported for some mycobacteria of medical interest, including *M*. *tuberculosis*, *Mycobacterium bovis* and *M*. *bovis* BCG [10], as well as for *M*. *ulcerans* and *Mycobacterium marinum* [9]. Also, the environmental *Mycobacterium smegmatis* has been investigated by using phenotypic PM1, PM3 and PM5 plates to test carbon and nitrogen sources and nutrient supplements [12]. In our study and previously reported studies, data were validated by the negativity of negative controls and the reproducibility of results over two wells per substrate. The protocol used in our study was adapted from the protocol used for studying *M*. *smegmatis* [12] whereas extensive modifications in preparing the inoculum for PM plates have been made over a previously reported protocol for studying *M*. *tuberculosis* [10]. In this report, the authors chose to investigate a *M*. *tuberculosis* Beijing clinical isolate instead of the *M*. *tuberculosis* H37Rv investigated by Biolog in a previously reported work [10]. Indeed, *M*. *tuberculosis* H37Rv is now cultured for 115 years [13] for approximately more than 50 passages in the authors’ laboratory; with a demonstrated genomic derive [14] that renders these plural *M*. *tuberculosis* H37Rv strains unrepresentative of the *M*. *tuberculosis* strains causing modern tuberculosis. Therefore, the results we obtained with *M*. *tuberculosis* used as a positive control must be tempered due to differences in methodology [10].

We observed that the *M*. *canettii* corebiologome (substrates metabolized by all the six *M*. *canettii* strains under investigation) was limited to only 17/190 (9%) carbon substrates and 3/95 (3%) nitrogen substrates. Searching for the potential sources common to these substrates highlighted plants as potential sources, but not fruits or vegetables. Our interpretation of these observations is that *M*. *canettii* may reside in close association with non-fruit bearing plants. This interpretation is reinforced by evidence for a potential relationship between *M*. *canettii* and cellulose, the major constituent of plant cell walls. Indeed, an unexpected conservation of cellulase-encoding genes has been found in the genome of *M*. *canettii* including the *Cel6* gene, which encodes a fully active cellulase [15].

The unique geographical specificity of *M*. *canettii* strains, which, apart from the three initial (lost) strains reported by G. Canetti himself, have all been isolated from patients exposed to one of the regions of the Horn of Africa [3], led us to the hypothesis that *M*. *canettii* lives in close association with one or several plants specifically endemic to this part of the world (Table 4). Moreover, populations must be in close contact with these potential plant reservoirs, either by using these plants for tissue preparation, medicinal and recreational activities or feeding. The fact that *M*. *canettii* is temperature-sensitive and specifically provokes lymph node infection along the digestive tract of patients [3] furthermore narrows the possibility to alimentary contacts with uncooked or poorly cooked plants [16]. Moreover, in this study, no significant association was found between *M*. *canettii* and sources related to aquatic environment (algae and mollusks), confirming that the potential plant reservoirs of *M*. *canettii* should be located in the arid environment in the Horn of Africa.

**Table 4:** List of plants endemic in countries of the Horn of Africa.

Country	Endemic plants	Use by local inhabitants	References
**Republic of Djibouti**	*Teucrium spicatum*		[19]
	*Phagnalon lavranosii*		[20]
	*Cynoglossopsis somalensis*		[21]
	*Matthiola puntensis*	Flowering plants	[21]
	*Livistona carinensis *	Leaves are used to cover theroofs	[22, 24]
	*Euphorbia godana *		[24]
	*Euphorbia amicorum*		[25]
	*Aloe ericahenriettae*		[24, 26]
	Aloe mcloughliniiChristian	**Medecine**: Laxative, soaking, crushed leaves or branches or stems in water for 12 h and the water is taken orally	[21, 27]
	*Caralluma mireillae*		[28]
	*Polygala goudahensis*		[21]
	*Taverniera oligantha *		[21, 29]
	*Volutaria djiboutensis*		[30]
	*Aloe djiboutiensis *		[26]
	Echidnopsis hirsuta		[31, 32]
	*Aponogeton nudifloris*	**Food**: Tuber consumption	[30]
	*Kalanchoe elliptica*		[30]
	*Farsetia longistyla*		[33]
	*Hildebrandtia somalensis*		[34–36]
**Ethiopia**	*Cordeauxia edulis*	**Food**: The seeds are eaten dried, boiled, roasted or raw. **Drinks**: People made a tea out of the leaves **Medicine**: *C*. *edulis* can regulate gastric secretion. A study showed that the consumption of the plant enhances the production of erythrocytes and is therefore used as a remedy for anemia.	[29]
	*Aloe mcloughlinii*Christian		[37]
	*Catha edulis*	Euphoric plant, recreational use	[38]
	*Echidnopsis hirsuta*		[32, 35]
	*Rhus glutinosa ssp abyssinica*	Species subject to strong animal pressure particularly when annual grasses disappear from pastures	[19]
	*Buxus hildebrandtii*	Used for the construction of traditional huts	[19, 21]
	*Farsetia longistyla*		[33]
	*Hildebrandtia somalensis*	The twigs are burned and the fumes are used by Borana women to purify and perfume their bodies and their clothes	[34–36]
	*Commiphora guidottii*	Source of sweet myrrh, use of the fragrant resin	[39, 40]
	*Echinops kebericho* Mesfin	Aromatic plant, medicinal plant with antimicrobial activities, treats fever, headache, stomachache, and cough	[41]
	*Thymus schimperi*	Aromatic plant, medicinal plant with antimicrobial activities	[41]
	*Thymus serrulatus*	Aromatic plant	[41]
	*Lippia adoensis *	Aromatic plant	[41]
	*Aframomum corrorima*	Aromatic plant	[41]
**Somalia**	*Cyclamen somalense *	Use of the fragrant resin	[35]
	*Cordeauxia edulis*	**Food**: The seeds are eaten dried, boiled, roasted or raw. **Drinks**: People made a tea out of the leaves. **Medicine**: *C*. *edulis* can regulate gastric secretion. A study showed that the consumption of the plant enhances the production of erythrocytes and is therefore used as a remedy for anemia.	[29]
	*Livistona carinensis *	Leaves are used to produce mats and baskets	[22, 23]
	Euphorbia amicorum		[25]
	*Dirachma somalensis*		[25]
	*Commiphora guidottii*	Source of sweet myrrh, use of the fragrant resin	[39, 40]
	*Echidnopsis hirsuta*		[32, 35]
	*Buxus hildebrandtii*	Used for the construction of traditional huts	[19, 21]
	*Kalanchoe elliptica*		[19]
	*Farsetia longistyla*		[33]
	*Hildebrandtia somalensis*		[34–36]

The names of plants common to the countries of the Horn of Africa are shaded green.

In this short list of plants, *Catha edulis* (known as khat) is fulfilling all these criteria as potential source for *M*. *canettii*. Khat, cultivated in Ethiopia and Eritrae, is then imported and circulating in Djibouti, Yemen and Somalia. Khat is specifically consumed as a stimulant or as a medicinal plant by local populations including children younger than 10 years in the Horn of Africa [17]. Interestingly, rhamnose, that we found to be one of the two substrates specifically metabolized by *M*. *canettii* but not by closely related *M*. *tuberculosis*, was detected in the leaves of khat [18].

## Conclusions

The original approach combining experimental study with a keyword study has been efficiently used to detect some ecological niches of *M*. *ulcerans*, a nontuberculous environmental mycobacterium [9]. Applying the very same methodology to *M*. *canettii* was also efficient in the present study, pointing towards some plants as potential ecological niches for this environmental tuberculous mycobacterium. Capitalizing on this new knowledge, further ethnobotanical studies will indicate plant usages by local populations, guiding field microbiological investigations in order to disclose one or several sources of infection for populations. In particular, plants could be screened by using PCR-based methods for the detection of DNA sequences specific for *M*. *canettii*, selecting plants to be further cultured. If these investigations confirm any association between *M*. *canettii* and khat or other plants, infection with *M*. *canettii* should be included in the very short list of phytonoses which are human infections transmitted by plants [42].
